# Beyond the Immunization Schedule: Unmasking Vaccine Failure in a Child With Down Syndrome

**DOI:** 10.1155/crpe/9928987

**Published:** 2026-06-19

**Authors:** Stacy B. Buchanan, Marissa Terry

**Affiliations:** ^1^ Nell Hodgson Woodruff School of Nursing, Emory University, Atlanta, Georgia, USA, emory.edu; ^2^ Children’s Healthcare of Atlanta, Atlanta, Georgia, USA, choa.org; ^3^ Loganville Pediatrics, Atlanta, Georgia, USA

**Keywords:** Down syndrome, immune dysregulation, pneumococcal-23 vaccine

## Abstract

Children with Down syndrome (DS) commonly experience recurrent infections and may have altered immune responses to routine vaccinations. Primary care providers routinely monitor typical DS‐associated comorbidities, but immunization effectiveness is not regularly assessed. Current guidelines recommend standard childhood vaccination schedules for children with DS, though evidence suggests some may have inadequate immune responses despite complete vaccination records. Here, we report the case of a 5‐year‐old child with DS who experienced frequent respiratory infections despite adherence to recommended immunizations and management of common comorbidities including cardiac disease, laryngomalacia, and gastroesophageal reflux disease. Following immunology consultation and administration of the 23‐valent pneumococcal vaccine, the frequency of respiratory infections notably decreased. This case highlights the importance of considering vaccine response when evaluating recurrent infections in children with DS, even when routine immunization records are complete. The management approach demonstrated how broadening the differential diagnosis beyond typical DS‐associated conditions and implementing additional immunization strategies can improve clinical outcomes. This case represents a possible approach that can inform shared decision‐making discussions between clinicians and families of children with DS regarding immunization monitoring and enhanced vaccination strategies.

**Article Summary.**
• A revelatory case of vaccine failure in Down syndrome challenges standard immunization protocols and presents a pathway to enhanced preventive care.

## 1. Introduction

Down syndrome (DS) is the most common chromosomal disorder, affecting approximately one in 700 live births in the United States [1]. While the physical and developmental characteristics of DS are widely recognized, the complex health needs of children with DS present ongoing challenges for healthcare providers. Primary care providers play a crucial role in managing the diverse medical issues associated with DS [2]. Among these challenges, the unique immunological profile of children with DS has emerged as an area of particular concern, especially regarding their response to vaccinations and susceptibility to infections.

The immune dysregulation in DS encompasses both innate and adaptive immune responses [1]. Studies have revealed alterations in T cell subsets, B cell populations, and cytokine production that may contribute to suboptimal vaccine responses [1, 3]. Of particular importance is the observed decrease in switched memory B cells in individuals with DS, which are crucial for mounting effective antibody responses to vaccines [4]. This immune dysregulation can significantly impact vaccine responses and increase infection susceptibility in pediatric patients, leaving them vulnerable to severe respiratory infections, as pneumococcal disease remains a significant cause of morbidity and mortality in this population [1, 5]. Given the potential for inadequate immune responses to routine vaccinations in children with DS, primary care providers must maintain a high index of suspicion for vaccine failure and subsequent infection risk. This case report aims to highlight the importance of monitoring vaccine responses and considering tailored immunization strategies in pediatric patients with DS. Here, we report the case of a 5‐year‐old boy with DS who experienced chronic respiratory illnesses due to an inadequate response to the pneumococcal‐23 vaccine.

## 2. Case Report

A 5‐year‐old boy with DS was referred to my pediatric practice for primary care management. At the initial presentation, he was in his usual state of health, which included ongoing speech and occupational therapy for developmental delays. He had a diagnosis of gastroesophageal reflux disease (GERD), and laryngomalacia requiring occasional albuterol use, but no other significant respiratory issues.

Several months post initial consultation, the patient began presenting with increased frequency to the clinic, primarily for upper respiratory symptoms. His typical clinical picture included persistent cough, sleep disturbances, and frequent progression to acute otitis media or rhinosinusitis. Initial management involved the use of an albuterol inhaler, which provided temporary relief from cough symptoms and mitigated sleep disruptions. When symptoms progressed, amoxicillin was typically prescribed with good clinical response noted.

The patient was born in the late preterm period at 36 6/7 weeks’ gestation via cesarean section. At birth, a murmur was noted and after consultation with a local pediatric cardiologist the following diagnoses were made, small apical muscular ventricular septal defect (VSD), mild tricuspid valve regurgitation, and left aortic arch with aberrant subclavian artery. Chromosomal testing sent after birth revealed DS as a primary diagnosis. Additionally, within the first few weeks of life, a diagnosis of laryngomalacia was made. Subsequently, this child developed GERD. As the child grew, developmental delay was noted in speech and fine motor skills for which he was receiving speech and occupational therapies.

His family history included maternal and sibling history of anemia and grandparental history of asthma, depression, dyslipidemia, and hypertension. The patient resided primarily with his mother under a shared custody arrangement, with regular care provided by biological grandparents. The family had reliable access to medical care and transportation.

The patient’s health began to deteriorate in late 2022 (see Table 1 for timeline of clinical events). In November, he was diagnosed with bronchitis, followed by pneumonia, neutropenia, and thrombocytopenia in December. During his 5‐year well exam that same month, his parents reported a recent hospitalization for hypoxia. Persistent symptoms of neutropenia and thrombocytopenia led to consultations with hematology–oncology and immunology in March of 2023.

**TABLE 1:** Timeline of clinical events.

2022‐11‐29	Diagnosed with bronchitis
2022‐12‐19	Diagnosed with pneumonia, neutropenia, thrombocytopenia
2022‐12‐28	5‐year well exam; anemia, neutropenia; parents discuss recent hospitalization for hypoxia
2023‐03‐10	Hematology–oncology and immunology consults
2023‐03‐21	Follow‐up via telephone; parent reports ED visit and hospitalization due to RSV bronchiolitis
2023‐04‐04	Clinic sick visit; URI; WBC 4.1, platelets 10.1
2023‐04‐24	Interval sick visit; bronchitis; prescribed cefdinir
2023‐05‐19	Interval office visit; URI, asthma exacerbation; prescribed nebulizer treatments and oral dexamethasone
2023‐05‐24	Office follow‐up; pulmonology and hematology follow‐up recommended
2023‐05‐26	Immunology consult visit; titers drawn
2023‐06‐09	Phone follow‐up; advised to return for pneumococcal 23 vaccine

As spring approached, the patient’s condition remained worrisome. His mother reported an emergency department visit and hospitalization due to RSV bronchiolitis in March. April and May brought continued upper respiratory infections, bronchitis, and symptoms of respiratory distress, necessitating treatments with cefdinir, nebulized medications, and oral dexamethasone.

Pulmonology was consulted, which led to a formal asthma diagnosis. Concurrently, immunology assessments, including titer tests, revealed an inadequate response to the pneumococcal vaccine. This finding prompted the administration of the 23‐valent pneumococcal vaccine in June. While ongoing evaluations for thrombocytopenia and neutropenia continued, the patient experienced a decrease in the frequency of upper respiratory infections throughout the following winter and spring.

## 3. Discussion

Children with DS face multiple health challenges that require vigilant monitoring and comprehensive care coordination. This case highlights both strengths and limitations in the management of recurrent infections in a child with DS. The initial approach appropriately followed American Academy of Pediatrics (AAP) guidelines for routine surveillance of common comorbidities, including cardiac disease, visual problems, and otitis media [2]. Chronic health needs may lead providers to overlook causes outside the commonly associated conditions. This patient had a history of laryngomalacia, asthma, and GERD, all potential contributors to frequent upper respiratory infections [1]. Less often, as in this case, dysregulated immunization response should be considered as a cause of frequent illness. The patient’s family was not vaccine hesitant, and the patient received all recommended immunizations, including the 13‐valent conjugate pneumococcal series. It was only after an immunology consult, inadequate vaccine response was considered. The 23‐valent polysaccharide pneumococcal vaccine was administered and the frequency of the patient’s upper respiratory infections declined. The causal relationship between enhanced immunization coverage and reduced infection frequency in this case, while promising, should be interpreted with appropriate caution given the natural variation in infection rates over time as well as underlying neutropenia. Nevertheless, the marked improvement in our patient’s health following intervention provides a compelling rationale for considering immune response monitoring in children with DS who experience recurrent infections.

The literature supports systematic screening for various comorbidities in children with DS, including thyroid dysfunction and hematologic disorders [2]. Recent studies have also documented altered immune responses in this population, though routine monitoring of vaccine titers is not yet part of standard guidelines [1–5]. Our patient’s improved clinical course following administration of the 23‐valent pneumococcal vaccine aligns with research suggesting additional immunization strategies may benefit children with DS who experience frequent infections despite routine vaccinations [5]. The standard vaccine schedule for children recommends a conjugate pneumococcal vaccine for all children aged 2 through 59 months old. For children 2 years and above with certain underlying health conditions, a polysaccharide pneumococcal vaccine should be administered [6]. For future consideration, additional conjugate vaccine boosters represent another protective strategy, as they link polysaccharides to protein carriers, enhancing immunogenicity through T‐cell‐dependent responses. This mechanism generates more effective, long‐lasting immunity and memory B cells [5, 7]. Research has shown that children with DS respond to conjugate vaccine boosters at levels comparable to their siblings, suggesting this approach could effectively address some of the immune deficiencies observed in this population [7].

This case emphasizes the importance of looking beyond standard approaches when providing healthcare for children with DS. While monitoring common comorbidities remains essential, providers must maintain a broader perspective, particularly regarding immune function and vaccine response. The implementation of enhanced immunization strategies, including antibody titer testing and conjugate vaccines, represents an evidence‐based approach that can be explored through shared decision‐making between healthcare providers and families. Through this collaborative framework, supported by specialist consultation when needed, we can work to optimize preventive care and improve outcomes for this vulnerable population.

## Author Contributions

Stacy B. Buchanan and Marissa Terry conceived and initiated this case report and contributed to its critical review and revision. Furthermore, both Stacy B. Buchanan and Marissa Terry collaborated on drafting the initial manuscript and contributed to its critical review and revision.

## Funding

No funding was received for this manuscript.

## Disclosure

Stacy B. Buchanan has worked on vaccine projects as a clinical expert through the National Association of Pediatric Nurse Practitioners along with the Centers for Control and Prevention, and Moderna. All authors have approved the final manuscript as submitted and agreed to be accountable for all aspects of the work.

## Consent

No written consent has been obtained from the patients as there are no patient identifiable data included in this case report.

## Conflicts of Interest

The authors declare no conflicts of interest.

## Data Availability

The data that support the findings of this study are available on request from the corresponding author. The data are not publicly available due to privacy or ethical restrictions.

## References

[1] Huggard D. , Doherty D. G. , and Molloy E. J. , Immune Dysregulation in Children With Down Syndrome, Frontiers in Pediatrics. (2020) 73, no. 73, 10.3389/fped.2020.00073. PMC7056667 32175298

[2] Bull M. J. , Trotter T. , Santoro S. L. , Christensen C. , and Grout R. W. , Health Supervision for Children and Adolescents With Down Syndrome, Pediatrics. (2022) 149, no. 5, 10.1542/peds.2022-057010. 35490285

[3] Malle L. , Patel R. S. , Martin-Fernandez M. et al., Autoimmunity in Down’s Syndrome via Cytokines, CD4 T Cells and CD11c+ B Cells, Nature. (2023) 615, no. 7951, 305–314, 10.1038/s41586-023-05736-y. 36813963 PMC9945839

[4] Carsetti R. , Valentini D. , Marcellini V. et al., Reduced Numbers of Switched Memory B Cells With High Terminal Differentiation Potential in Down Syndrome, European Journal of Immunology. (2015) 45, no. 3, 903–914, 10.1002/eji.201445049. 25472482 PMC4674966

[5] Santoro S. L. , Baloh C. H. , Hart S. J. et al., Pneumonia Vaccine Response in Individuals With Down Syndrome at Three Specialty Clinics, American Journal of Medical Genetics. (2023) 193, no. 4, 10.1002/ajmg.c.32070. 37864360

[6] Valentini D. , Marcellini V. , Bianchi S. et al., Generation of Switched Memory B Cells in Response to Vaccination in Down Syndrome Children and Their Siblings, Vaccine. (2015) 33, no. 48, 6689–6696, 10.1016/j.vaccine.2015.10.083. 26518399

[7] Kimberlin D. W. , Banerjee R. , Barnett E. D. , Lynfield R. , and Sawyer M. H. , Red Book: 2024–2027 Report of the Committee on Infectious Diseases, 2024, 33rd edition, American Academy of Pediatrics, 10.1542/9781610027373.

